# Supplementary material for: Skimming genomes for systematics and DNA barcodes of corals
Source: Ecol Evol. 2024 May 13;14(5):e11254. doi: 10.1002/ece3.11254 (PMC11091489; doi:10.1002/ece3.11254)
Supplement: Supplementary file 2 — Appendix S2. [file ECE3-14-e11254-s001.pdf]

DNA CTAB Extraction Protocol for EtOH-Preserved Specimens

1. Blot sample to remove excess EtOH. Place tissue in ~1.0 mL 2X CTAB in a 1.5 mL microfuge tube. Let **soak for ~24 hrs**, changing the CTAB buffer several times. (Put samples on "Nutator" rotary platform to mix them gently during this period.)

2. Add 0.2% (1.0 μL) β-mercaptoethanol (βME) to 500 μL 2X CTAB in a 1.5 mL Eppendorf tube and incubate at 65°C for 5 min.

3. Remove a sample from CTAB, blot to remove excess liquid, place it in a ceramic mortar and cover it immediately with liquid nitrogen. Use pestle to pulverize tissue to a fine powder while it is still frozen. Immediately scrape powdered tissue into the tube containing warm CTAB/βME.

4. Add 5 μL proteinase K (20 mg/mL), mix gently by inverting tube several times, and incubate at 65°C for 1-4 hrs (or at 55°C for 24 hrs for very old specimens). Flick tube periodically to mix.

5. Centrifuge tube for several seconds and gently transfer liquid supernatant to a clean 1.5 mL tube. Discard pellet.

6. Add ~600 μL PCI (25:24:1 phenol:chloroform:isoamyl alcohol) and mix gently for 10 min. Centrifuge at top speed (13,000 rpm) for 5 min at 4°C.

7. Transfer top (aqueous) layer to a clean 1.5 mL tube, being careful not to disturb the interface. (If the interface is very thick or poorly formed repeat step 6, using ~500 μL PCI.)

8. Chill on ice for 20 min.

9. Add 500 μL of cold chloroform and 100 μL Phytopure resin. Mix gently for 10 min, then centrifuge at 1200 rpm for 10 min at 4°C.

10. Transfer top (aqueous) layer to clean 1.5 mL tube. Add an equal volume of cold isopropanol. Chill in -20°C freezer overnight. (Samples can be left in freezer indefinitely at this stage.)

11. Centrifuge for 30 min at 4°C. Drain off ethanol (invert tube or use pipette).

12. Add 1.0 mL cold 70% ethanol and centrifuge 10 min at 4°C. Drain off ethanol. Repeat.

13. Dry pellet in SpeedVac (~25 min without heat) or air-dry by suspending tube upside down.

14. Add 25 μL sterile TE buffer and resuspend DNA by gently flicking and inverting tube.

15. Add1.25 μL RNase (1.0 mg/mL stock solution; final concentration in reaction should be 50 μg/μL) and incubate at 37°C for 30 min.

Extraction Reagents

2X CTAB Extraction Buffer:

140 mL 5M NaCl
20 mL 0.5M EDTA
25 mL 2M tris-HCl, pH 8.0
10 g CTAB

Milli-Q H_2O to 500 mL
Autoclave.

Store at room temperature.

TE Buffer: 10 mM tris-Cl (pH 7.4 or pH 8.0)
1 mM EDTA (pH 8.0)

Phenol: Equilibrate to pH>7.8 and store under 100mM Tris-HCl pH 8.0 in light-tight bottle. Add hydroxyquinoline to a final concentration of 0.1% as an antioxidant. To prolong shelf life to >6 months store at -20°C.

Re-precipitation of DNA
Add 1/10 volume 3M NaAcetate and 2.5 volumes of cold 100% EtOH.
