# Supplementary material for: Skimming genomes for systematics and DNA barcodes of corals
Source: Ecol Evol. 2024 May 13;14(5):e11254. doi: 10.1002/ece3.11254 (PMC11091489; doi:10.1002/ece3.11254)
Supplement: Supplementary file 3 — Appendix S3. [file ECE3-14-e11254-s003.pdf]

Appendix S3

Skimming genomes for systematics and DNA barcodes of corals

Andrea M. Quattrini[1], Luke J. McCartin[2], Erin E. Easton[3], Jeremy Horowitz[1], Herman H. Wirshing[1], Hailey Bowers[1], Kenneth Mitchell[4], María del P. González-García[1,5], Makiri Sei[1], Catherine S. McFadden[4], Santiago Herrera[1,2]

[1] Department of Invertebrate Zoology, National Museum of Natural History, Smithsonian Institution, 10th St. & Constitution Ave. NW, Washington, D.C. 20560, United States
[2] Department of Biological Sciences, Lehigh University, Bethlehem, PA 18015
[3] School of Earth, Environmental, and Marine Sciences, University of Texas Rio Grande Valley, Port Isabel, TX 78597
[4] Department of Biology, Harvey Mudd College, Claremont, CA 91711
[5] Department of Marine Sciences, University of Puerto Rico, Mayagüez, PR 00681

Corresponding Author: Andrea M. Quattrini, quattrinia@si.edu

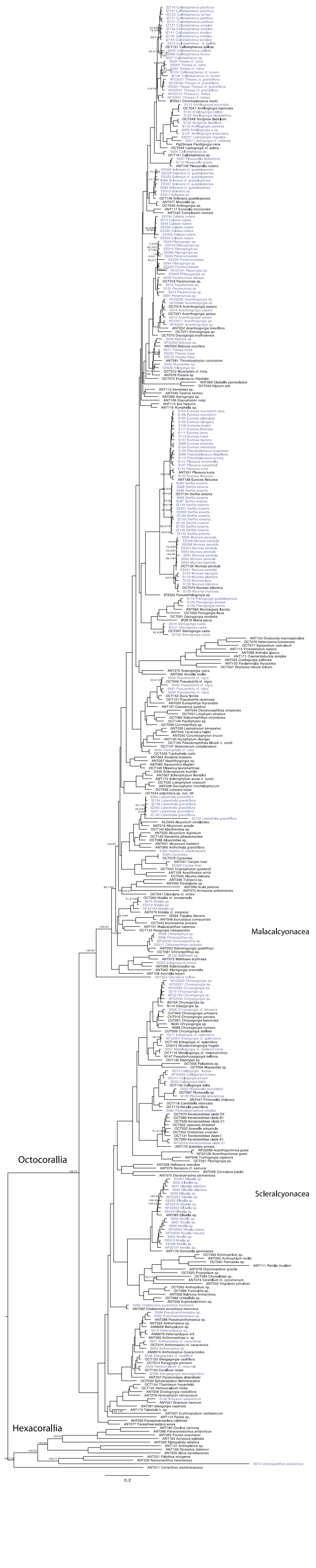

Supplemental Figure 1. Maximum-likelihood phylogenetic tree of Octocorallia based on UCEs and exons with branch lengths shown. Tree rooted to Hexacorallia. Tip labels in purple=genome skim, black=target capture. Ultrafast bootstraps and Sh-alRT values both >95% unless indicated.

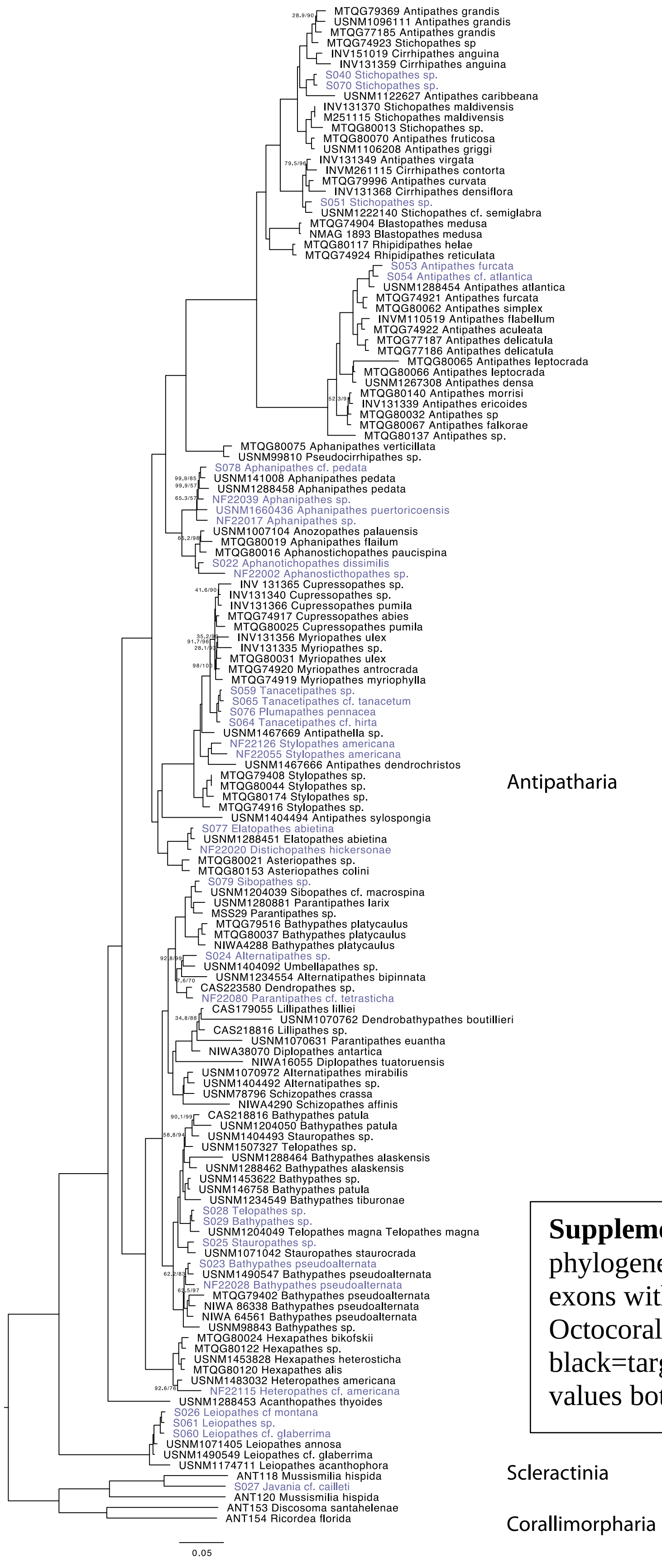

Supplemental Figure 2. Maximum-likelihood phylogenetic tree of Antipatharia based on UCEs and exons with branch lengths shown. Tree rooted to Octocorallia. Tip labels in purple=genome skim, black=target capture. Ultrafast bootstraps and Sh-alRT values both >95% unless indicated.

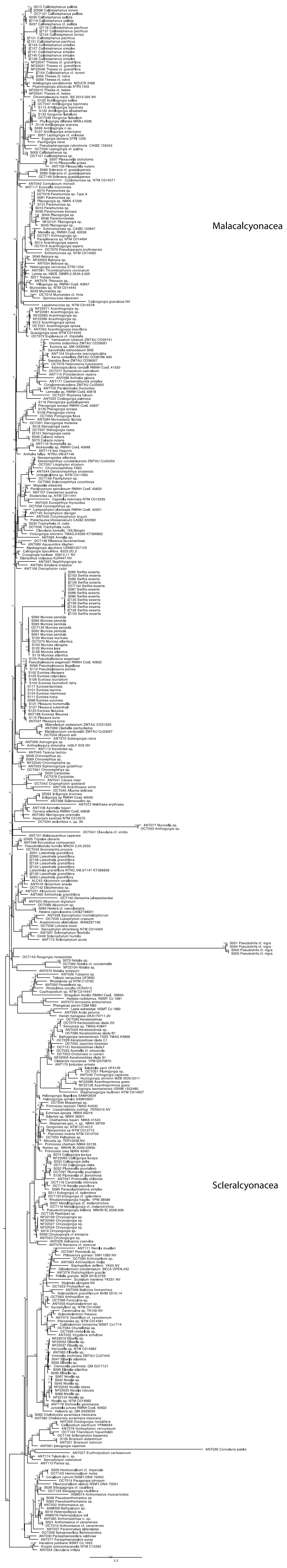

Supplemental Figure 3. Maximum-likelihood phylogenetic tree of *mtMutS* sequences (1,074 bp alignment) generated from genome skimming data and PCR/Sanger Sequencing (McFadden et al. 2022). The Scleralcyonacea is rooted to the Malacalcyonacea. Ultrafast bootstraps are included.

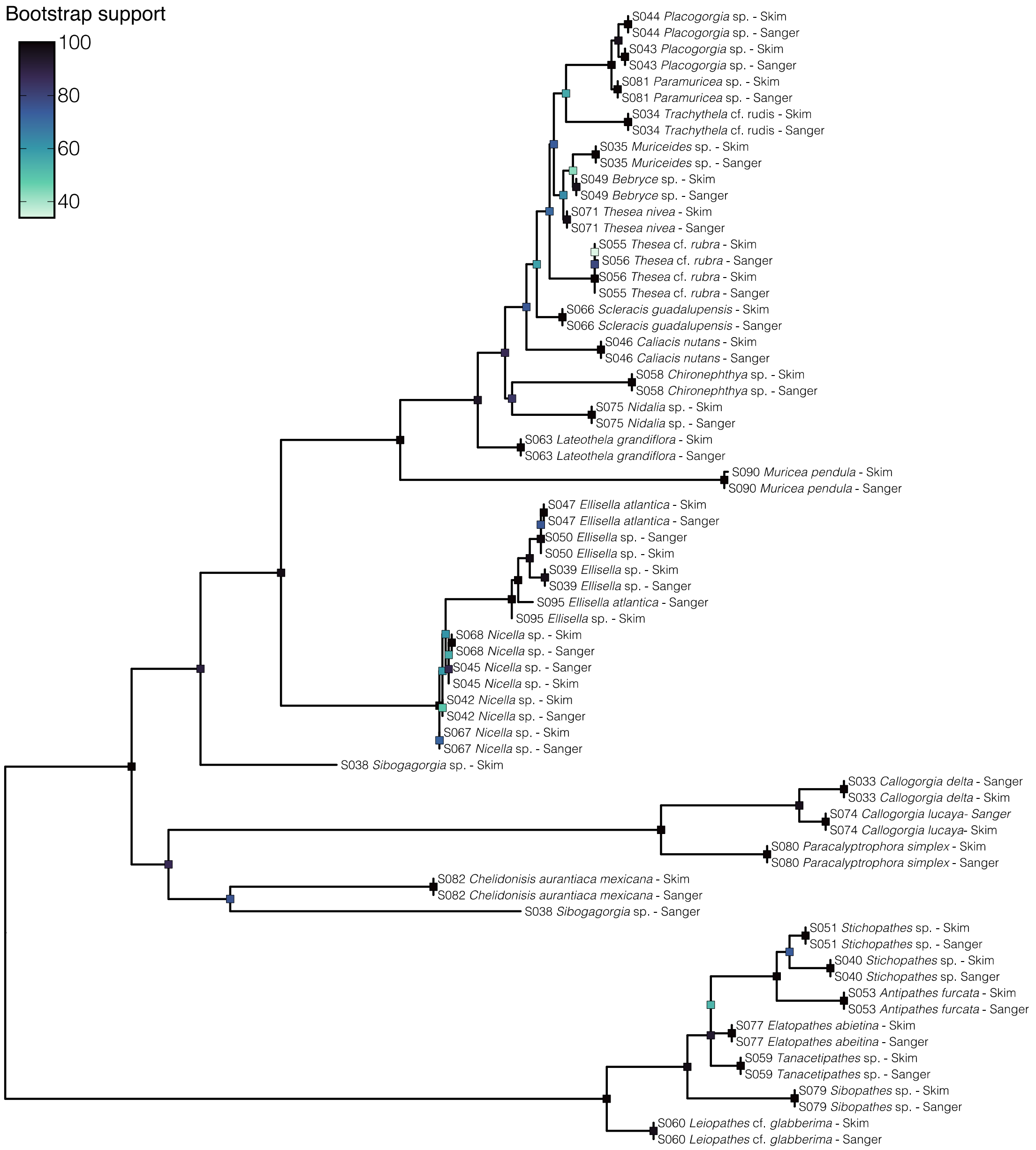

Supplemental Figure 4: Maximum-likelihood phylogenetic tree of *28S rRNA* barcode sequences (478 bp alignment) generated from 35 samples via conventional PCR and Sanger sequencing and from genome skimming data. The tree is rooted at its midpoint. Ultrafast bootstraps are included.

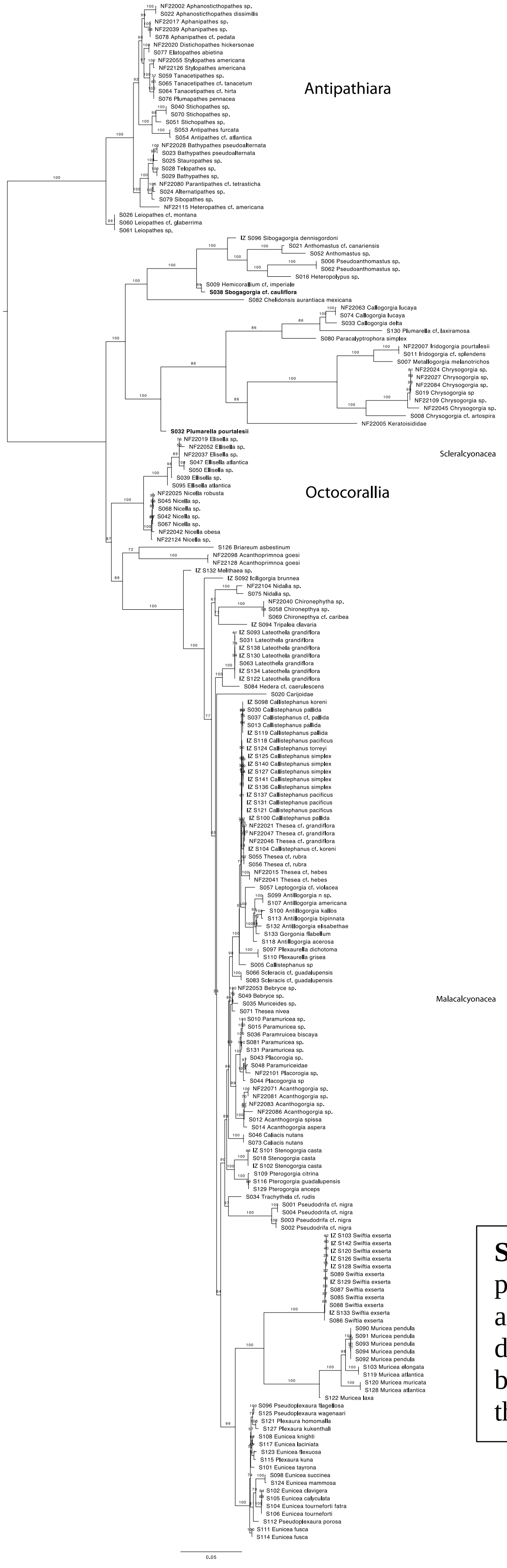

Supplemental Figure 5. Maximum-likelihood phylogenetic tree of *rRNA* gene sequences (6,031 bp alignment) generated from genome skimming data. The tree is rooted at its midpoint. Ultrafast bootstraps are included. Specimens in bold are those that are in aberrant positions in the tree.
